# The relationship between aggressive behaviors of preschool children and the violence against Iranian women in the COVID-19 pandemic

**DOI:** 10.1186/s12905-022-01954-0

**Published:** 2022-10-05

**Authors:** Neda Asadi, Fatemeh Salmani, Mahin Salmani

**Affiliations:** 1 Nursing Research Center, Kerman University of Medical Sciences, Kerman, Iran; 2 Nursing & Midwifery Sciences Development Research Center, Najafabad Branch, Islamic Azad University, Najafabad, Iran; 3 Department of Mathematics and Statistics, University of New Brunswick, Fredericton, Canada

**Keywords:** Children, Aggressive, Behaviors, Violence, Coronavirus, Pandemic

## Abstract

**Background:**

During epidemics, supports are limited and individual and collective vulnerabilities as well as domestic violence are increased. Therefore, various groups in society, especially children and their mothers, are extremely vulnerable. This study aimed to assess the relationship between aggressive behaviors of preschool children and the violence against Iranian women during the COVID-19 pandemic.

**Methods:**

This descriptive-correlational study was conducted in October–November 2020. Stratified random sampling was performed among preschool children in Kerman. Data were collected using the Violence toward Women Inventory and the Aggression scale for preschoolers Scale. Data were analyzed using SPSS25, ANOVA, independent t-test, and Pearson correlation test.

**Results:**

The results showed that the total mean scores of violence against women and preschoolers’ aggression were 54.43 ± 10.6 and 88.44 ± 6.5, respectively. The results showed a statistically significant difference in aggressive behaviors of preschool children, mother's job, number of children, mother's education, income, and age. A positive and significant relationship was also found between the subscales of violence against women and aggression in preschool children.

**Conclusions:**

The results showed a positive and significant relationship between violence against women and aggression of preschool children. Therefore, it is recommended that parents identify and eliminate the risk factors for domestic violence during the COVID-19 in order to protect their children. Parents also must learn coping strategies for stress and resilience in the epidemic crises.

## Background

Corona Virus Disease 2019 (COVID-19) has been declared a global pandemic in the twenty-first century [1]. Despite measures taken to prevent the spread of the disease, they have adverse effects, including physical and mental health risks, isolation and loneliness, closure of schools and businesses, and economic vulnerabilities. Domestic violence is one of the physical and mental health risks in this crisis [2, 3]. Domestic violence refers to threatening behaviors in families that may be physical, sexual, psychological, or economic and includes intimate partner violence [4]. Violence against women has both an impact on their physical and mental health and on children's social skills and behaviors. Parents' mental and behavioral states are very effective in young children [5]. It is a common misconception that children in preschool ages are unaware of violence. According to research, any early exposure to violence is part of a child's worldview [6]. Children who do not have a safe domestic place are hidden victims of domestic violence. Domestic violence can have devastating consequences for children, including aggressive behaviors [3, 7].

Social isolation policies during epidemics limit supports and increase individual and collective vulnerabilities as well as domestic violence. Therefore, various groups in society, especially children and their mothers, are extremely vulnerable [2]. Evidence shows an unprecedented increase in violence against women and children around the world since the beginning of social isolation and lockdown [4, 8, 9]. For example, there was a 40–50 point increase in domestic violence in Brazil during the lockdown [10]. Mothers’ domestic violence is associated with a variety of individual and social factors [11]. Certain cultures and laws can play an important role. Therefore, the prevalence of domestic violence in developing countries is more common than it is in developed countries because of economic crises, poverty, gender gap between men and women, and difficult living conditions in these countries [7].

Many Iranian mothers are victims of various forms of domestic violence. According to Moazen et al. [12], 54.5 percent of the Iranian women are victims of domestic violence [12]. Given the impact of social, economic and cultural factors on the incidence of domestic violence, it seems inevitable to study the effects of this social problem on women and their children during the COVID-19 pandemic.

Violence against mothers of preschool children can have very destructive physical and psychological effects on these children, which can make people prone to delinquency or various physical and mental diseases in adulthood [5]. Since the researchers in the present study could not find a similar study, they decided to investigate the relationship between aggressive behavior in preschool children and violence against Iranian women during the COVID-19 outbreak. By identifying the destructive consequences of violence against women, the government and organizations can pave the way for effective measures to avoid this abnormal social phenomenon, both during and after the outbreak of emerging diseases. Lack of accurate and timely information may increase the risk of psychological and social problems in this vulnerable group.

### Hypothesis

The hypothesis of this study was that there was a relationship between violence in women and aggression of preschool children during the COVID-19 pandemic.

## Methods

### Design and ethical consideration

This descriptive correlational study was designed to identify the relationship between aggressive behaviors of preschool children and the violence against Iranian women during the COVID-19 pandemic.

The ethics committee of Kerman University of Medical Sciences approved the study with the project No. 99000232 and the code of ethics No. IR.KMU.REC.1399.305. Participation in this study was voluntary. The study goals and procedures were explained to all participants, and the mothers’ informed consent was obtained.

### Sample and setting

This study was conducted on mothers of preschool children in Kerman (southeast of Iran) during 2 months (mid-October–mid-November 2020).

**Fig.1:**
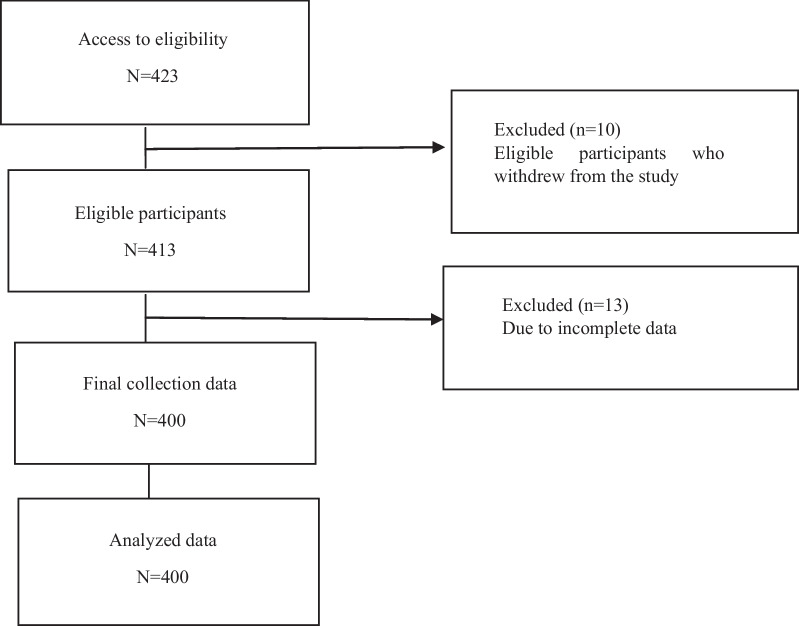
Flow chart of the Study

Stratified random sampling was used to select 117 governmental and non-governmental preschool schools from five districts in Kerman (five from each district). Four hundred and twenty-three children were selected from 25 schools using a random number table. Then, their mothers, who had inclusion criteria, were contacted and included in the study. Out of 423 mothers, 10 did not want to enter the study. Finally, 413 mothers with preschool children joined the WhatsApp group 13 of whom were excluded from the study because they did not completed the questionnaires perfectly (Fig. 1).

Inclusion criteria were the ability to read and write in Persian and access to social media and the Internet. Non-response to more than one third of the questionnaire was the exclusion criterion. Cochran's formula with infinite population was used to calculate the sample size. *Z* = 1.96, *p* = *q* = 0.5, and *d* = 0.03 were considered. Therefore, 384 participants were estimated for the present study. By taking into account a ten-percent dropout, 423 subjects were estimated.

This study was conducted in Kerman in southeastern Iran. In addition, the level of violence against women in Kerman was not studied.

### Measures

The researchers uploaded the electronic form of the questionnaires in WhatsApp groups. Demographic and background information questionnaire, violence toward women inventory and aggression scale for preschoolers were used in this research. Violence toward Women Inventory (VTWI) includes 32 items and 4 subscales: psychological violence (1–16), physical violence (27–17), sexual violence (28–30) and economic violence (31–32). The inventory is rated on a 3-point Likert scale (never = 1, once = 2, twice or more = 3). The total score is between 32 and 96. The total scores for the subscales of psychological, physical, sexual and economic violence are 16–48, 11–33, 3–9 and 2–6, respectively. Cronbach's alpha of this questionnaire is 0.97 [13, 14].

Items of psychological violence include verbal aggression, insults, defamation, intimidation, blaming thoughts and ideas. Physical violence may include slapping, pushing, throwing objects, breaking objects, attacking, strangling and pulling hair. Sexual violence may include sexual dissatisfaction, insults and humiliation during sexual intercourse, and forced sexual intercourse. Economic violence may include not complying with economic responsibilities, such as alimony, being stingy and threatening while spending.

Ghorbani et al. (2022) in Iran validated and measured psychometrically this tool [15]. In the present study, the reliability of the instrument was investigated and Cronbach's alpha of the total instrument was estimated to be 0.89.

Aggression scale for preschoolers contains 42 items and includes relational, physical and reactive-verbal aggression rated on a five-point Likert scale (never = zero, rarely = 1, once a month = 2, once a week = 3, most days = 4). The score of this questionnaire is between 0 and 168, with higher score indicating a high level of aggression in children.

This questionnaire evaluates various types of aggression, including verbal aggression, physical aggression, and behavioral aggression (swearing, insults, threatening, bullying, throwing others out of the game, nervousness, intolerance of failure, beating, breaking others’ belongings, arguing,, indifference).

Cronbach’s alpha coefficient of the whole scale has been 0.98 [16]. In the present study, the reliability of the instrument was investigated and Cronbach's alpha of the total instrument was estimated to be 0.91.

### Data analysis

First, *Kolmogorov–Smirnov test* was used to check the normality of the data, so descriptive (frequency, percent, mean and standard deviation) and inferential statistics (*Pearson correlation, independent sample t-test and one-way ANOVA*) and SPSS25 were used. Significance level was considered 0.05 (*p* ≤ 0.5).

## Results

Results of the study showed that 55.7% of the preschoolers were female, 48.3% of the families had two children, 49.3% of the mothers had bachelor's degree and 54.8% of the mothers were employed. More than half of the participants had a monthly income of 100–200 US dollars (average) (Table 1).

**Table 1:** Demographic characteristics of the study samples and their associations with violence against women and aggressive behaviors of preschool children

Characteristics		n(%)	Violence against women		Aggressive behaviors of preschool children	
			Mean (SD)	*p*	Mean (SD)	*p*
Gender of the child	Girl	233(55.7)	52.4(7)	*t* = − 1.67 *p* = 0.094	86.38(6.7)	*t* = − 0.177 *p* = 0.86
	Boy	177(44.3)	54.2(12.7)		86.4(6.3)	
Mother's job	Employed	219(54.8)	56.3(13)	*t* = 6.22 *p* < 0.001	87.17(6.5)	*t* = 2.49 *p* = 0.013
	Housewife	181(45.2)	49(0.9)		85.6(6.3)	
number of children	1	113(28.2)	54.7(14)	*F* = 3.2 *p* = 0.02	86.5(6.8)	*F* = 34.77 *p* < 0.001
	2	193(48.3)	52(8.8)		84.15(4.7)	
	3	81(20.3)	55.6(9.2)		90.37(6.7)	
	More than 3	13(3.3)	50(1)		96(1)	
Mother's education	High school	60(15)	60.2(16.9)	*F* = 14.42 *p* < 0.001	88.8(7)	*F* = 5.88 *p* = 0.001
	Diploma	67(16.8)	52(4.2)		88(7)	
	Bachelor	197(49.3)	51.6(9.2)		85.5(6.1)	
	others	60(15)	52.42(4.5)		85.6(5.5)	
Family income per month	Under 100 US dollars	93(23.3)	55.50(12.2)	*F* = 9.9 *p* < 0.001	87.3(6.3)	*F* = 6.17 *p* = 0.002
	100–200 US dollars	221(55.3)	50.65(4.3)		86(6.9)	
	Above 200 US dollars	86(21.5)	51.12(9.7)		84.6(5.9)	
Mother's age	< 25	17(4.3)	53(1)	*F* = 10.77 *p* < 0.001	93(1)	*F* = 37.65 *p* < 0.001
	26–30	77(19.3)	52.12(3.9)		83.7(3.6)	
	31–35	153(38.3)	52(12.6)		84.1(5.6)	
	36–40	126(31.5)	53.3(7.8)		88.3(6.9)	
	> 40	27(6.8)	65.6(16.7)		96(3.9)	

The results of *independent t-test* showed no statistically significant relationship between violence against women and the gender of the child. While violence against women was a statistically significant correlation with the mother's job, it was higher among employed women. In addition, the results of the *one-way analysis of variance* showed a statistically significant correlation between violence against women, the number of children, mother's education, income, and age, with women with 3 children, high school degree, income under 3 million and mothers over 40 years old experiencing more violence.

The results of *independent t-test* showed no statistically significant difference between aggressive behaviors of preschool children and the child's gender. While aggression in preschool children had a statistically significant correlation with mother's job, it were higher among employed mothers. In addition, the results of *one-way analysis of variance* showed a statistically significant correlation between aggression in preschool children, number of children, mother's education, income, and age. Preschool children aged over 3 years old whose mothers were over 40 years old, had high school degrees and incomes under 3 million showed higher aggressive behaviors. (Table 1).

The results of the study showed that the overall mean of violence against women was 54.43 ± 10.6 during the COVID-19 pandemic, with the mean scores for psychological violence, physical violence, sexual violence, and economic violence being 28.7 ± 8.9, 20.6 ± 7.7, 4.3 ± 1.8 and 2.7 ± 1.2, respectively (Table 2).

**Table 2:** Mean score of violence against women and subscales of psychological, physical, sexual and economic violence

Group variable	Mean	Standard deviation
Psychological violence	28.7	8.9
Physical violence	20.6	7.7
Sexual violence	4.3	1.8
Economic violence	2.7	1.2
Violence against women	54.4	10.6

Results of the study showed that the mean score of preschoolers’ aggression was 88.44 ± 6.5 during the COVID-19 pandemic.

Pearson correlation coefficient showed a positive and significant relationship between the total scores of violence against women and preschoolers’ aggression, indicating that the higher the violence against women, the higher the aggression in preschool children (*r* = 0.33, *p* < 0.001). A positive and significant relationship was also found between the subscales of psychological (*r* = 0.39, *p* < 0.001), physical (*r* = 0.33, *p* < 0.001), economic (*r* = 0.015, *p* < 0.001) violence and aggression in preschool children, implying that with the increase of psychological, physical and economic violence against women, aggressive behaviors increased in preschool children. However, there was no significant relationship between sexual violence against women and aggressive behaviors in preschool children (Table 3).

**Table 3:** The correlation between scores of violence against women and aggression in preschool children

Variable	Aggression score	
	Pearson correlation coefficient	*p *-value
Psychological Violence	0.39	< 0.001
Physical Violence	0.33	< 0.001
Sexual Violence	0.015	0.76
Economic Violence	0.127	0.01
Violence Against Women	0.33	< 0.001

## Discussion

This study aimed to investigate the relationship between aggressive behaviors of the preschoolers and violence against Iranian women during the COVID-19 pandemic. According to this study, the average level of violence against women was greater among women with a bachelor's degree and a low income. Many studies supported the results of the present study and indicated that low level of education and socioeconomic status were associated with more verbal and physical violence against women [17, 18]. However, some studies showed no difference between education levels, income, and violence against women [19, 20]. The differences between these studies and our study may be due to different sample sizes or cultures of the study population.

The present study showed a higher rate of violence among children and older women, while some studies showed that the incidence of violence was higher among young mothers due to lack of experience and sufficient skills in problem solving [19]. In addition, Ghazanfari et al. [21] showed the rate of violence against women in women over 45 years old and in areas with a higher population density [21], which is consistent with the present study. Older women experience greater emotional and psychological changes due to the approach of menopause and hormonal changes, making them more prone to violence. Violence against mothers is also higher in larger families due to socioeconomic problems.

According to the present study, the rate of violence among employed women was higher than among housewives; although, no study in this area was found. The reason may be that employed women experience more problems and fatigue due to working outside and doing housekeeping; therefore, they are not patient at home and experience violence against women.

The results of the study showed the high rate of psychological and physical violence against Iranian women during the COVID-19 pandemic. Vakili et al. [22] also found the highest rate of psychological and physical violence among women in southern Iran [22]. Studies conducted in Brazil [23] and the United Kingdom [24] showed that psychological and physical violence against women was higher than other dimensions of violence, which is consistent with the present study.

Kassim [7] demonstrated that after African countries, the rate of violence against Asian women was higher [7]. The studies mentioned above examined violence against women during non-epidemic periods, but they were included in this study because there were few comparable studies. According to Malathesh et al. [25], stress caused by physical and economic constraints, business closures, unemployment, and a lack of social support were all factors that increased violence against women in India during the COVID-19 pandemic [25]. In addition, Sánchez et al. [26] reviewed 38 articles and found that some factors increased violence against women during social distancing [26]. Gebrewahd et al. [27] in Ethiopia also found an increase in psychological and physical violence against women during the COVID-19 pandemic [27]. Reports from China and the United Kingdom also show a threefold rise of violence against women and an increase in female mortality during the COVID-19 pandemic [28], which are consistent with the present study. The present study also showed that families experienced tensions due to social and physical distancing during the COVID-19 pandemic, which provided opportunities for violence against women.

The results of the present study also showed that the mean score of preschooler’s aggressive behaviors was moderate during the COVID-19 pandemic (88.44 ± 6.5). Children are direct or indirect victims of domestic violence [29]. Marchetti et al. [30] showed an increase in abnormal behaviors of the children during the COVID-19 epidemic. Verbal aggression and hyperactivity have increased in children for various reasons. This study considered such behaviors as a result of psychological distress in parents, violence against mother and child [30]. English et al. [31] also showed that mistreatment and intimate partner violence increased verbal aggression in children [31].

Platt et al. [32] in southern Brazil found that domestic violence increased due to social isolation during the COVID-19 pandemic. They showed that child ignorance, physical, psychological, sexual violence, and child labor increased during the COVID-19 pandemic, which led to abnormal behaviors in children, such as aggression. This study is consistent with the present study [33]. In addition to housework and childcare, increased health measures for women, restrictions, financial constraints and general insecurity have increased psychological stress in women, which can affect children's aggression [34]. On the other hand, spending of 24 h with children and closure of schools, parks and places for leisure time, no interaction with close family members such as grandparents, relatives and friends during the COVID-19 pandemic, cause stress, loss of motivation, and aggression in children [32].

The study results showed that with the increase of violence against women, aggressive behaviors increased in preschool children. There was also a positive and significant relationship between subscales of psychological violence, physical violence, economic violence and aggressive behaviors in preschoolers, implying that as psychological, physical and economic violence against women increased, so did aggressive behaviors in preschool children. Studies show that violence against women exposes children to physical and sexual abuse. Forty percent of the individuals, who witness domestic violence, are abused physically especially by mothers, who have been exposed to physical or psychological violence. However, the mother herself can be the savior of the physically abused child [34]. On the other hand, a physically or psychologically abused preschooler is more likely to be at risk of physical violence, aggression and delinquency during childhood and adolescence [35].

Violence against women exists in all countries of the world and is influenced by cultural factors, socio-economic class, age, education, income, ethnicity, and environmental conditions. Violence has prolonged devastating effects on the survivors even after the violence is over [36].

COVID-19 disease has had a detrimental effect on the environmental and familial conditions of individuals, resulting in verbal and physical conflicts between families during lockdown and long hours together. Family disputes, which are sometimes due to psychological and economic pressures related to this disease, have led to physical or psychological violence against women and children [33]. Pu et al. [37] showed that the high coexistence of partner violence and child physical abuse caused all kinds of aggression in children [37]. Capaldi et al. [38] also showed that children exposed to domestic violence and negative parenting behaviors had a lower level of childhood skills [38]. As a result, controlling negative emotions, external behaviors, interacting with peers, and focusing on learning skills are all difficult for these children. Such risks have devastating consequences, leading to uncontrollable and aggressive behaviors in children [39]. Mothers, who have experienced violence, are less sensitive to their children’s emotional needs and exhibit negative behaviors toward them. Children's learning, reactions to others, and expectations are all influenced by parental relationships [40, 41].

The current study had several limitations. It was a descriptive study, and the questionnaires were completed online due to COVID-19 pandemic, which could reduce the accuracy of the study. In addition, the relationship between violence against women and aggression of preschool children was not reported in the non-COVID-19 period, and it is impossible to establish with certainty whether these effects are related to the COVID-19 period. Therefore, further research in non-COVID-19 period is suggested to compare the results.

## Conclusion

The results of the study showed aggression among preschool children of the mothers exposed to physical, psychological and economic violence during the COVID-19 pandemic. As a result, it is recommended that parents identify and eliminate the risk factors for domestic violence during the COVID-19 in order to protect their children. Parents also must learn coping strategies for stress and resilience in the epidemic crises. It is also suggested that policymakers and governments pay special attention to vulnerable members of society, such as women and children, during pandemics, and that they protect families from the stressors caused by these diseases through various initiatives.

## Data Availability

The data are available upon request to the corresponding author after signing appropriate documents in line with ethical application and the decision of the Ethics Committee.
